# Experimental Investigation of the Solid Conveying Behavior of Smooth and Grooved Single-Screw Extruders at High Screw Speeds

**DOI:** 10.3390/polym14050898

**Published:** 2022-02-24

**Authors:** Kai S. Johann, Stephan Mehlich, Marcus Laichinger, Christian Bonten

**Affiliations:** Institut für Kunststofftechnik, University of Stuttgart, 70569 Stuttgart, Germany; mehlichstephan@yahoo.de (S.M.); laichinger.marcus@outlook.de (M.L.); christian.bonten@ikt.uni-stuttgart.de (C.B.)

**Keywords:** extrusion, solid conveying, grooved feed zone, non-linear throughput, high screw speed, varying granule geometry, analytical calculation

## Abstract

Single-screw extrusion at high screw speeds is established nowadays since it allows for a high mass throughput at a comparatively small extruder size. Compared to conventional extrusion at low screw speeds, a considerable non-linearity in mass throughput appears by exceeding a certain threshold screw speed. In this study, the solid conveying behavior of different plastic granules with varying geometries was investigated in a smooth, a helically and an axially grooved solid conveying zone for screw speeds up to 1350 rpm. These experimental findings are compared to classical analytical predictions in the literature. It is found for the first time that both the shape and size of the plastic granules play a decisive role in determining the threshold screw speed at which a non-linear mass throughput is observed. It is shown that small and spherical granules exhibit a later onset of non-linear throughput compared to larger lenticular and cylindrical shaped granules. Moreover, it is revealed that the mass throughput equalizes for an axially and a helically grooved solid conveying zone at high screw speeds. This is contrary to the low screw speed range where the axially grooved barrel results in a significantly higher throughput than the helically grooved barrel. Thus, the maximum throughput at high screw speeds is limited by the granule stream provided by the hopper opening and is no longer governed by the groove angle.

## 1. Introduction

### 1.1. Particularities of Single-Screw Extrusion at High Screw Speeds

Single-screw extrusion is one of the most important processes in plastics industry. It allows for the mass production of semi-finished products such as foils, plates, tubes and other rather simple profiles [1]. Classical single-screw extruders consist of a three-zone screw and a smooth barrel. This setup was extended 1959 by combining a barrier screw with a smooth barrel [2,3]. In the USA, both principles are still frequently used. In Europe, the development of extruders with a grooved feed zone began around the same time. The basic idea was to prevent the plastic granules from spinning with the screw due to the increased friction on the barrel wall. The intention was to achieve an increase in mass throughput and to stabilize the process through block flow. A continuous pressure build-up over the process length is characteristic for smooth barrel extruders. Contrary, in grooved barrel extruders the significant pressure build-up already occurs in the feed zone [1]. The subsequent melting and metering zones then act as pressure consumers. Therefore, in contrast to smooth barrel extruders, the mass throughput of grooved barrel extruders does not depend on the flow resistance of the die [1]. Attempts to combine barrier screws with grooved feed zones were carried out around 1980 [3,4]. Since then, these systems have been continuously further developed in terms of increasing the specific throughput and by increasing the screw speeds [5,6,7,8].

High-speed single-screw extruders are becoming more and more important since the end of the 1990s [9,10]. The first definition of what is considered “high-speed” was made at the end of the 1970s, where a circumferential screw speed of more than 1 m/s was defined as a criterion for demarcation [11]. However, in 2010 it was suggested for not taking the circumferential screw speed as criterion for demarcation. Instead, a screw speed of 400 rpm was proposed, since extruders with large screw diameters exceed the value of 1 m/s already at quite low screw speeds [12]. In general, high-speed operation is expected to result in high throughputs with small machine sizes and lower motor power [12]. Nevertheless, existing systems partly reach their limits in terms of throughput stability, sufficient melting capacity, homogeneity of the extrudate and melt temperature [13]. High screw speeds also have a strong impact on the feeding behavior, leading to throughputs falling short of expectations.

To investigate the influence of different screw designs on the decrease in specific throughput at high screw speeds, various screws with different flight pitch and depth were analyzed in [14], each with a similar free volume. It was found that the specific throughput drops less steeply for lower flight pitches and for higher flight depths, which is valid for almost all granules investigated [14]. In [15], the influence of the feed opening size and the dumping height above the feed zone was investigated, whereby the latter had no measurable effect on the throughput. In contrast, a decreasing feed opening width resulted in a decreasing specific throughput [15]. The length of the feed opening and its shape were modified in [16]. There, higher specific throughputs were achieved with an increasing feed opening length. The geometrical feed opening shape was altered between circle, parallelogram and square, whereby the circular shape ensured the highest throughputs [16]. However, this was attributed to the fact that the circle had the largest axial length of all opening geometries with the same area. A new compression design in the feed zone was investigated in [17]. The new compression section allowed for an alignment of the specific throughput of regrind and virgin material. This can be regarded as an important progress, because both throughputs commonly strongly differ in present extrusion systems since regrind materials usually exhibit significantly lower bulk densities [17].

The suitability of the commercially available Helibar^®^ extrusion system, which consists of a helically grooved feeding and plasticizing zone in combination with a barrier screw, was also already examined for high-speed operation [18,19]. The aim of this research was to use the advantages of such continuously grooved system, because they are characterized by a high pressure and conveying stability as well as a low melt temperature even at high back pressures [20,21]. In these investigations throughputs of up to 400 kg/h were achieved with different polyolefins using a 35 mm diameter screw, while a drop in specific throughput was observed with increasing screw speed [18,19].

This phenomenon at high speeds is also well known for smooth barrel systems [22]. There are various explanations which usually assume a partial filling of the first screw channels when the conveying capacity exceeds the feed flow provided by the hopper. An associated effect is the occurrence of granule vortex formation respectively recirculation at the end of the feed opening which reduces the effective feed opening length [23]. Nowadays, the decreasing specific throughput is partly compensated by locally providing a larger inner barrel diameter below the feed opening, so-called feed pockets. An enlargement of the feed opening thus counteracts the drop in specific throughput [10,16]. In [24], constructive approaches in screw design were investigated to reduce the decrease in specific throughput at high screw speeds. The focus was on preventing any circulating movement of the granule. On the one hand, it was found that an increasing screw pitch along the feed opening increases the throughput compared to a constant pitch [24]. On the other hand, a decreasing pitch proved to be unsuitable for increasing the throughput. An approach of using baffles to reduce the granule vortex formation proved to be unsuccessful [24]. In [25], the assumption that the screw channels are partially filled with granule as the speed increases was verified both experimentally and simulatively. Furthermore, it was found that the respective filling degree depends on the acting counter pressure [25].

### 1.2. Analytical Calculation of the Mass Throughput in a Grooved Solid Conveying Zone

Schneider [26,27] investigated the influence of solid conveying on the throughput behavior of smooth barrel extruders. He concluded that the friction conditions between the inner barrel wall and the material conveyed decisively influence the throughput. However, his mathematical formulation to predict the throughput is only successful if the actual friction coefficients, pressure distribution and pressure propagation are known. Goldacker [28] took up these formulations using a plastic powder and confirmed the assumptions made by Schneider. In addition, he also took the effect of a grooved barrel into account by defining an average friction coefficient from external and internal friction components. Therefore, this enabled first throughput calculations for grooved barrel systems.

Peiffer [29] developed an approach for calculating the throughput in grooved barrel extruders. He assumed an increasing friction in the barrel due to grooves filled with plastic granules. Nevertheless, an additional mass flow in the grooves was neglected. Grünschloß [30] developed a calculation approach which considers an additional mass flow in the grooves. Furthermore, he took into account, that the actual bulk density is significantly smaller than the bulk density that is usually determined at high dumping heights due to wall effects within the extruder. Grünschloß also assumed fully filled screw channels and that the plastic granule is conveyed as a block, the so-called nut-screw conveying. This allows to regard the solid conveying angle as equivalent to the groove angle, which facilitates the calculation substantially. Otherwise, the solid conveying angle must be calculated by solving the force and moment balance equation based on the pressure and friction forces acting on the solid granule bed. However, the latter requires the precise knowledge of the governing friction coefficients [29,31].

For grooved barrel extruders, Schöppner [31] distinguished four different solid conveying cases labeled as 1.(a), 1.(b), 2.(a) and 2.(b). The distinction depends on the relation between the mean granule diameter *d*_gra_ to the groove depth *h*_g_ and to the screw channel depth *h*_s_. The first case 1.(a) is equivalent to the case of Grünschloß and assumes a block flow in the groove direction, if the screw channel depth is *smaller* than two times the mean granule diameter and if the groove depth is smaller than the mean granule diameter. The second case 1.(b) is a mixed case, which assumes slipping between the granule layer in the grooves and the granule layer in the screw channel. This results in a different axial conveying velocity in the grooves and in the screw channel. The case 1.(b) is assumed to take place if the screw channel depth is *larger* than two times the mean granule diameter and if the groove depth is smaller than mean granule diameter. The third case 2.(a) and the fourth case 2.(b) apply if the screw channel depth as well as the groove depth are larger respectively significantly larger than the mean granule diameter. In contrast to the cases 1.(a) and 1.(b), both cases 2.(a) and 2.(b) presume that there is no additional mass flow in the grooves [31,32]. The two latter cases nevertheless consider a friction increase to the barrel due to the filled grooves, which is resembling to the considerations of Peiffer.

For predicting the mass throughput $\dot{m}$ in the feed zone as a function of the screw speed *n*, the following linear approach is frequently applied [32]:

(1) $$\dot{m}=v_{\mathrm{ax}}\cdot A_{f}\cdot \rho_{b}\cdot f$$

With $v_{\mathrm{ax}}$ being the axial conveying velocity of the solid plastic granule directly depending on the screw speed, $A_{f}$ being the free channel cross-sectional area of the chosen extruder, $\rho_{b}$ being the bulk density of the plastic granule and *f* being the filling degree, which is assumed to be equal one at low screw speeds.

The axial conveying velocity $v_{\mathrm{ax}}$ can be calculated by Equation (2) which contains the circumferential speed $v_{\mathrm{Circ}}$, the helix angle of the screw φ as well as the solid conveying angle α. The circumferential speed depends on the outer screw diameter $D_{s}$ and on the screw speed:

(2) $$v_{\mathrm{ax}}=v_{\mathrm{Circ}}\cdot \frac{\tan\varphi }{\frac{\tan\varphi }{\tan\alpha }+1}=\pi \cdot n\cdot D_{s}\cdot \frac{\tan\varphi }{\frac{\tan\varphi }{\tan\alpha }+1}$$

The free channel cross-sectional area of the extruder can be determined according to Equation (3), whereas the first term reduces the free cross-sectional area by subtracting the flight area. The second term is equivalent to the free cross-sectional area of the grooves and thus disappears in smooth barrels.

(3) $$A_{f}=\left(\frac{\pi }{4}\cdot \left(D_{s}^{2}-D_{c}^{2}\right)-\frac{w_{f}}{\sin\varphi }\cdot h_{s}\cdot i_{s}\right)+\left(\frac{w_{g}}{\sin\omega }\cdot h_{g}\cdot i_{g}\right)$$

In Equation (3) $w_{f}$ is the width of the flight, $w_{g}$ is the width of the groove, $D_{c}$ is the core diameter of the screw, $h_{s}$ is the channel depth, $h_{g}$ is the groove depth, ω is the groove angle, $i_{s}$ is the number of screw flights in the cross-sectional area, and $i_{g}$ is the number of grooves. The total mass throughput in a grooved feed zone thus comprises the throughput in the screw channel as well as the throughput in the grooves. 

In order to consider the fact that the bulk density of the plastic granule is actually smaller within the extruder compared to the bulk density at high dumping heights, Grünschloß [30] formulated an approach to fit measured bulk densities at different dumping heights $h_{\mathrm{du}}$:

(4) $$\rho_{b}\left(h_{\mathrm{du}}\right)=\rho_{0}\cdot \left[1-exp\left\{-A\cdot {\left(\frac{h_{\mathrm{du}}}{h_{0}}-1\right)}^{B}\right\}\right]$$

where $\rho_{0}$ is the maximum bulk density at high dumping height, $h_{0}$ is the dumping height threshold value (for $h_{\mathrm{du}}<h_{0}\;$ the bulk density equals zero) and *A* and *B* are fitting parameters without physical meaning.

Further references regarding the description of the solid conveying zone can be found in a review article of Wilczyński et al. [33]. Furthermore [33] addresses the modeling of the melting zone, the melt conveying zone and the coupling of all zones to enable global modeling of extruders.

It is known that the classical linear approach in Equation (1) fails to predict the mass throughput if a certain threshold screw speed is exceeded and thus only applies at low screw speeds. This phenomenon was already subject of different studies in the past, which consider the solid conveying at high screw speeds for smooth barrel extruders [34,35,36].

In contrast to smooth barrel extruders, there is a significant lack of systematic experimental data of the solid conveying behavior in extruders with grooved feed zones, particularly concerning high screw speeds. Thus, the objective of this work is to thoroughly study the solid conveying behavior in a smooth as well as in a helically and an axially grooved feed zone at high screw speeds up to 1350 rpm for the first time. Since the granule geometry is expected to play a decisive role in the solid conveying behavior, three different types of plastics and three different granule shapes are examined. The obtained data will be compared to the classical linear throughput predictions by assuming a nut-screw conveying based on [30]. For attaining a proper throughput prediction, the dependency of the bulk density on the dumping height is considered in the calculations. This reveals potential starting points for developing a new analytical approach for predicting the throughput of grooved single-screw extruders even at high screw speeds, which will be the aim of further work.

## 2. Materials and Methods

**Figure 1:**
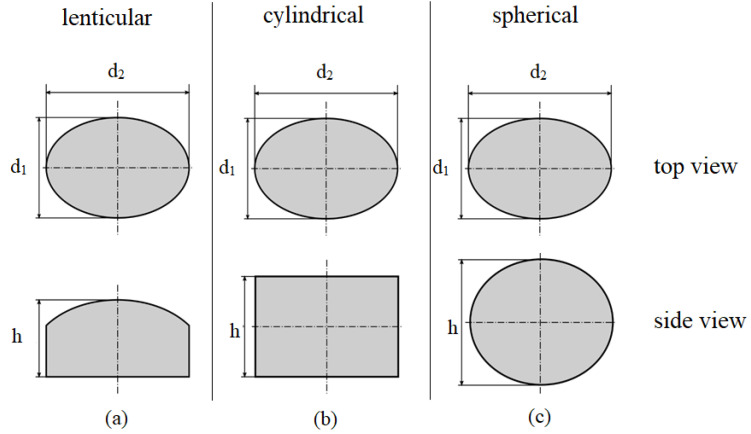
Schematic representation of (**a**) the lenticular, (**b**) the cylindrical and (**c**) the spherical granule shapes and dimensions.

Three different types of plastics were chosen, whereas each type exhibits two different granule geometries. The selected high-density polyethylene (PE-HD) is *Hostalen CRP 100 Black* from the company LyondellBasell, Rotterdam, the Netherlands, which is a highly viscous extrusion type that can be used in pipe extrusion processes. The chosen polypropylene (PP) homopolymer is *Moplen HP400H* from LyondellBasell, Rotterdam, the Netherlands, which is, according to its data sheet, typically used in extrusion blow molded bottles. Besides these two polyolefins, *Ultramid B40L* from BASF SE, Ludwigshafen, Germany, was selected as a polyamide 6 (PA) which can be used for sheet extrusion. PE-HD and PP were chosen since polyolefins are the most common plastics and successfully processed with grooved barrel extruders. PA was chosen to investigate the effects of a material with distinctly different friction characteristics. Both the virgin PE-HD as well as the virgin PP exhibit a lenticular granule geometry, whereas the virgin PA has an almost spherical shape. Since the PA granule is not perfectly spherical, the diameter was measured at three different positions (see Table 1). The three granule shapes are simplified depicted in Figure 1 with two diameters *d*_1_ and *d*_2_ as well as the height *h*.

In order to obtain an additional grain shape, which is identical for all three materials, a small lab extruder (30/25D) from the Collin Lab & Pilot Solutions GmbH, Maitenbeth, Germany, equipped with a round-hole nozzle with 2.8 mm diameter was used to extrude the respective materials. The extruded strand was cut with a pelletizer unit to obtain cylindrical granules. Due to an imperfect pull-off procedure and gravitational effects the obtained cylindrical granule was not perfectly round, which is schematically shown in Figure 1.

Since the geometrical dimensions may affect the solid conveying behavior and the vortex formation in the hopper at high screw speeds, a hundred random grains for each type of granule were measured with regards to its height and its two diameters. The equivalent spherical diameter (ESD) was calculated for classifying the granules based on the four solid conveying cases according to Schöppner, which were previously elucidated. Since doubling the ESD results in values larger than the screw channel depth and since the ESD is larger than the groove depth case 1.(a) can be assumed for five granule types. The only exception is the spherical PA, where case 2.(a) applies, because the ESD is smaller than the groove depth. Furthermore, the average grain mass was measured by weighing a hundred grains for each type (see Table 1).

The bulk density was determined as a function of the dumping height according to the approach of Grünschloß [30]. The measuring cup exhibited a 50 mm diameter and a height adjustable bottom. The bulk density was determined for dumping heights between 2–20 mm in 2 mm steps and for a dumping height of 50 mm according to DIN EN ISO 60 [37]. Each measurement was conducted three times with the mean values being depicted in the following chapter.

**Figure 2:**
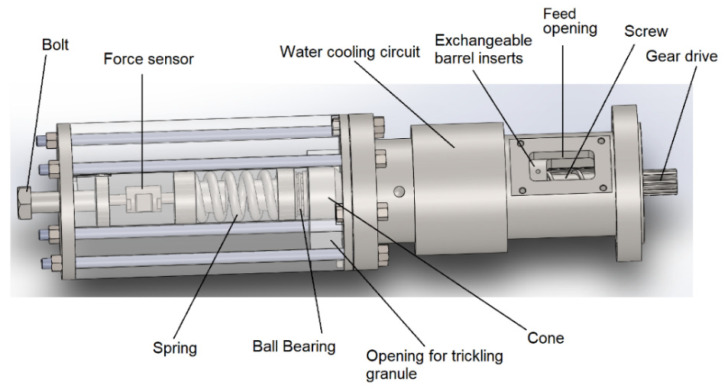
Computer-aided design (CAD) model of the experimental extrusion set-up (mere solid conveying zone) with the mounted back pressure element on the left-hand side.

The experimental investigations were primarily focused on analyzing the solid conveying behavior in the mere feed zone and thus melting of the plastic should be avoided. Hence, an experimental setup was developed that only includes the solid conveying zone of a common extruder without further zones. The setup shown in Figure 2 consists of a water-cooled outer steel casing. This allows to plug in different types of steel barrels with an overall length of 300 mm, an outer diameter of 70 mm and an inner diameter of 35 mm. One smooth, one helically and one axially grooved barrel were used. For both grooved barrels the grooves have a continuous depth of 2.8 mm in the 80 mm long feed opening and then continuously diminish to zero along the residual length of 220 mm. Further geometrical information regarding the number, angle and width of the grooves as well as information concerning the relevant screw geometry can be found in Table 2.

This experimental setup enables solid conveying without a back pressure that counteracts on the conveying capacity because the granule can trickle out freely after exiting the feed zone. For all mass throughput measurements, the granule exiting the feed zone was collected for 45 s at a certain screw speed and subsequently weighed. The measurements showed a high reproducibility with a maximum deviation of 5% between independent series of tests. This deviation comes primarily from the human reaction time of putting a vessel below the trickle-out area and removing it immediately after the measurement time.

For studying the additional effect of a back pressure that usually acts upon the conveyed granule in real extrusion processes, a back pressure element was designed based on previous works [35] possessing own adaptions. The back pressure element could be easily mounted upon the feed zone setup and exerted a force upon the exiting plastic granule via a tensioned spring. The spring is connected to a cone that is pressed against the trickling gap with a vertical trickle-out area of 582 mm^2^. The bolt at the left end enabled the progressive tensioning of the spring and the force sensor recorded the applied axial force. The ball bearing ensured that the rotational movement of the screw respectively the cone is neither transferred to the spring nor to the force sensor.

**Figure 3:**
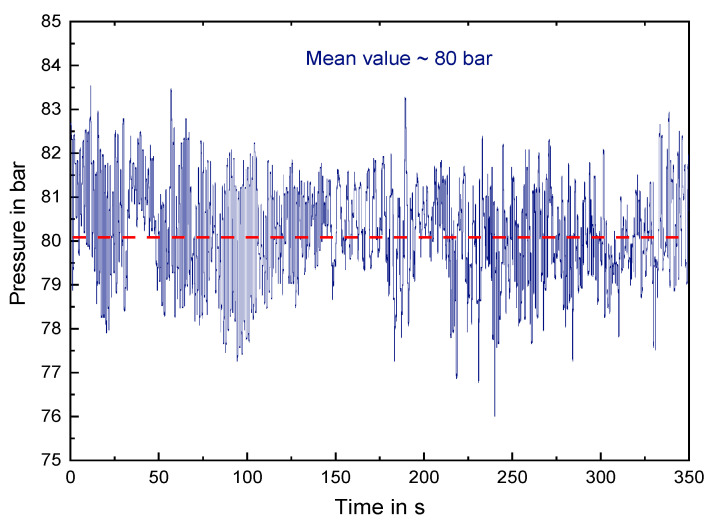
Oscillating pressure profile of the designed back pressure element.

Figure 3 illustrates that the force recorded via *LabVIEW* from National Instruments Corporation, Austin, Texas, USA, respectively the calculated pressure oscillated rapidly over time. This was accompanied by a fast horizontal back and forth hammering of the simultaneously rotating conus. The back pressure values given in Chapter 3 are thus mean values with a standard deviation of around ±2 bar. In the following work only the mean values of the back pressure are denoted for simplicity. The maximum spring force of the utilized spring is 8.600 N. This theoretically allows applying up to 150 bar. Nevertheless, due to safety reasons and to avoid possible set-up damage, the maximum applied pressure was set to 80 bar.

**Figure 4:**
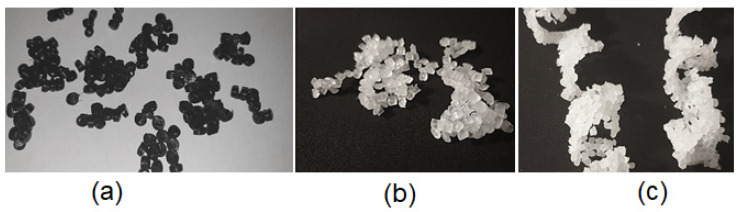
Partial clumping of (**a**) PE-HD, (**b**) PP and (**c**) PA granules when using a grooved barrel despite utilizing an additional water cooling.

It was observed that despite intense water cooling both grooved barrels led to a partial clustering of the granule particles. This clustering emerged even in the freely trickling case without the mounted back pressure element in a certain screw speed range, which varied for the PE-HD, PP and PA. Since these solid particle clusters (see Figure 4) lead to a plugging of the narrow trickling gap, only the smooth barrel could be equipped with the back pressure element.

In addition, the determined mass throughput of the mere solid conveying zone was compared to an entire extrusion set-up, including melting of the solid granule as well as outflow of the polymer melt through a die. Therefore, the lenticular PE and the lenticular PP were processed using a complete extruder set-up (35/34D). This entire extruder set-up possesses a throttle die, which allows for the adjustment of the flow area within the die and thus for adjusting the die back pressure (see Chapter 3.5). Increasing the die back pressure also leads to an overall pressure increase in the whole barrel. Two kinds of barrel were used: one barrel with a helically grooved solid conveying zone and a subsequent smooth barrel and one barrel which is smooth from end-to-end. In both cases, the geometry of the barrel and of the screw is identical to the geometries of the mere solid conveying zone experiments. For the grooved barrel extruder and for the full-length smooth extruder the solid conveying zone was cooled down to around 60 °C, whereas the residual zones and the die were set to 240 °C. 

## 3. Results and Discussion

### 3.1. Calculation of the Mass Throughput via a Linear Approach Assuming Nut-Screw Conveying

First, the mass throughput for each conducted experiment is predicted by using the linear approach of Equation (1). To do so, the free channel cross-sectional area of the respective extrusion barrel is calculated by Equation (3) using the geometry values given in Table 2. The corresponding free channel cross-sectional area is 443.83 mm^2^ for the smooth barrel, 584.14 mm^2^ for the helically grooved barrel and 597.83 mm^2^ for the axially grooved barrel. Obviously, both grooved barrels possess an enlarged free cross-sectional area compared to the smooth barrel. The groove geometries were previously chosen to exhibit nearly the same free cross-sectional area which thus only differs by around 2% between the helically and the axially grooved barrel. 

Determining the solid conveying angle to calculate the axial conveying velocity usually requires information about the systems governing friction coefficients. Since the experimental determination of the friction coefficients plastic-plastic, plastic-screw and plastic-barrel is error-prone, a nut-screw conveying according to Grünschloß is assumed for all used materials. This results in equalizing the solid conveying angle with the groove angle with ω=α=41.19° for the helically and ω=α=90° for the axially grooved feed zone (cf. solid conveying case 1.(a)). This cannot be assumed for the smooth barrel and hence a solid conveying angle of 25° was assumed as an empirical value for the smooth solid conveying zone. However, it should be kept in mind that the latter assumption is a strong simplification, because varying the type of plastic granule results in different friction coefficients and thus in different solid conveying angles respectively different axial conveying velocities.

**Figure 5:**
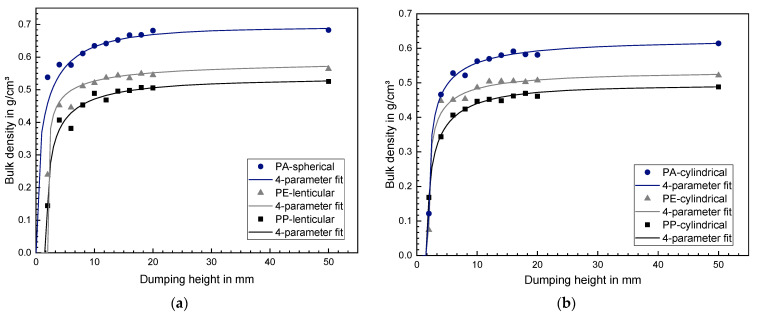
(**a**) Bulk density as a function of dumping height for spherical PA, lenticular PE and lenticular PP (**b**) Bulk density as a function of dumping height for cylindrical PA, cylindrical PE as well as cylindrical PP.

The results of the bulk density determination for different dumping heights are shown in Figure 5a for the virgin granules and in Figure 5b for the cylindrical regrind. All six granule types first exhibit a significantly increasing bulk density with increasing dumping height and slowly approach a maximum value by exceeding approximately 10 mm dumping height. The cylindrical granules possess a smaller bulk density at higher dumping heights compared to the respective virgin granule analogue. For both cases, PA exhibits the highest bulk density, followed by PE and subsequently followed by PP. Equation (4) was used to fit the measured data, whereas the fitting parameters are shown in Table 3. Since the screw channel depth is 5.5 mm, the associated fitting functions were used to calculate the bulk density at a dumping height of 5.5 mm. These values are between 15–32% smaller than the bulk densities at a dumping height of 50 mm for all investigated materials.

Combining Equations (1)–(3) and using in the respective geometrical values leads to the following equations:

(5) $${\dot{m}}_{smooth}=20.75\;\mathrm{mm}\cdot 443.83\;2{\mathrm{mm}}^{2}\cdot \rho_{b}\left(h_{\mathrm{du}}=5.5\;\mathrm{mm}\right)\cdot n$$

(6) $${\dot{m}}_{helical\;grooves}=25.61\;\mathrm{mm}\cdot 584.14\;{\mathrm{mm}}^{2}\cdot \rho_{b}\left(h_{\mathrm{du}}=5.5\;\mathrm{mm}\right)\cdot n$$

(7) $${\dot{m}}_{axial\;grooves}=34.95\;\mathrm{mm}\cdot 597.83\;{\mathrm{mm}}^{2}\cdot \rho_{b}\left(h_{\mathrm{du}}=5.5\;\mathrm{mm}\right)\cdot n$$

In these three cases fully filled screw channels are assumed, meaning *f* equals one. The equations only depend on the bulk densities of the six different granule types and on the screw speed. It is obvious that the highest mass throughput for a certain type of granule and a certain screw speed is to be expected for the axially grooved barrel. This is due to the significantly higher axial velocity compared to the smooth and the helically grooved barrel. In addition, both grooved barrels exhibit a higher free cross-sectional area in which solid conveying can proceed, compared to the smooth barrel.

### 3.2. Results of the Solid Conveying Behavior of PE-HD Granules

**Figure 6:**
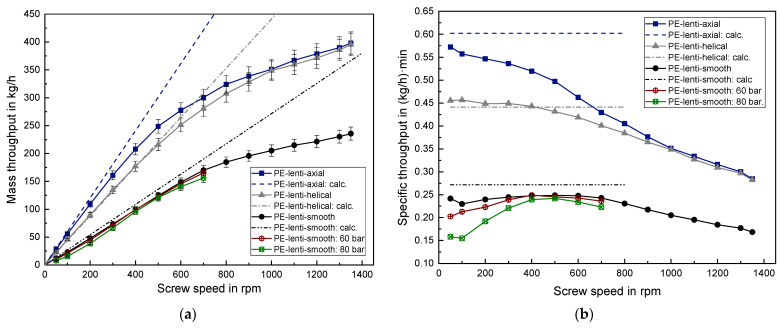
(**a**) Absolute mass throughput and (**b**) specific mass throughput for lenticular PE.

The results of the mass throughput determination of the lenticular PE-HD are shown in Figure 6 for three different barrel types and for two applied back pressures. Figure 6a depicts the absolute mass throughput as a function of screw speed from 50 rpm (revolutions per minute) up to 1350 rpm. The circumferential screw speed thus varies between 0.09 m/s and 2.47 m/s. The dashed straight lines are the calculated mass throughput based on the Equations (5)–(7). Figure 6b shows the specific throughput, which describes the throughput per screw rotation. Examining the specific throughput facilitates to assess the linearity at low screw speeds, represented by a horizontal in the specific throughput diagram.

All measured curves in Figure 6 exhibit an approximately linear behavior at lower screw speeds. However, after exceeding a certain threshold screw speed, a non-linear respectively degressive behavior can be observed. This is accompanied by slowly approaching a maximum value. At low screw speed, the axially grooved barrel results in a significantly higher mass throughput compared to the helically grooved barrel. This is due to the higher solid conveying angle and the higher axial conveying velocity. The difference in mass throughput between the axially and helically grooved solid conveying zone diminishes by increasing the screw speed. Moreover, when exceeding a screw speed of 800 rpm the mass throughput completely converges, taking into account the standard error of the measurements. Since the smooth barrel possesses no additional free cross-sectional area of the grooves that can contribute to a mass transfer as well as it has a smaller solid conveying angle, the obtained mass throughput is roughly two times smaller in this case compared to the grooved barrels.

Mounting the back pressure element onto the solid conveying zone with a smooth barrel and applying a back pressure of 60 bar and 80 bar, respectively, results in a small mass throughput decrease in the case of 60 bar and a significant decrease in the case of 80 bar, particularly at low screw speeds. Nevertheless, by exceeding 300 rpm the measured values nearly equalize. Whether this latter observation is caused by real effects or by operational uncertainties of the back pressure element must be evaluated in future work.

By examining the mass throughput prediction based on the simple linear law in Equation (1) it becomes obvious that this approach is only reasonable up to around 300 rpm. For the range between 50–300 rpm the mean relative deviation between the calculated mass throughput and the measured mass throughput is approximately 14% for the smooth barrel, 9% for the axially grooved barrel and only 2% for the helically grooved barrel.

**Figure 7:**
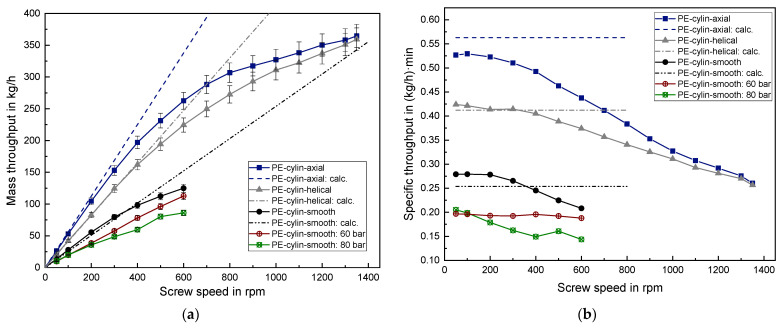
(**a**) Absolute mass throughput and (**b**) specific mass throughput for cylindrical PE.

The results of the cylindrical PE-HD are shown in Figure 7. Qualitatively the same behavior is observed for both lenticular PE-HD and cylindrical PE-HD, namely a convergence of the throughput curves at higher screw speed, when using the axially and helically grooved barrel. Furthermore, no significant difference between lenticular and cylindrical PE-HD can be observed regarding the threshold screw speed, despite both granules differ in geometry as well as in the average grain mass with 28.6 mg for the cylindrical and 35.2 mg for the lenticular PE-HD granule.

Quantitative comparison of the cylindrical PE-HD and the lenticular PE-HD always reveals a lower mass throughput for the cylindrical PE-HD for the whole screw speed range. The maximum value at 1350 rpm using lenticular PE-HD is about 400 kg/h, whereas using the cylindrical PE-HD results in a maximum value of around 360 kg/h. This observation also applies for the low screw speed range between 50–300 rpm. Here, the lenticular PE-HD possesses a 5–8% higher mass throughput for the axially grooved barrel, a 7–8% higher throughput for the helically grooved barrel and a 7–13% higher throughput for the smooth barrel, always compared to the cylindrical PE. This can mainly be explained by the bulk density difference at a dumping height of 5.5 mm which is about 7% higher in the lenticular PE-HD case (see Table 3).

Utilizing a smooth barrel and lenticular PE limited the maximum screw speed to 600 rpm since further increase led to an intolerable noise generation due to friction. The applied back pressure of 60 bar and 80 bar led to a considerable reduction of mass throughput for all examined screw speeds.

Comparing the calculated throughput with the measured throughput of cylindrical PE reveals a proper prediction accuracy. The relative deviation is around 8% in the case of the smooth barrel, around 8% in the case of the axially grooved barrel and only 1% in the case of the helically grooved barrel, considering the low screw speed range of 50–300 rpm.

Again, the calculation of the axially grooved barrel tends to overestimate the throughput. This reveals that the assumed solid conveying angle of 90° is probably somewhat lower in reality. Despite the solid conveying angle of 25° is just a simplified assumption in the case of the smooth barrel, the prediction accuracy is surprisingly well. The very low deviation of the predicted values to the measured values in the helically grooved case reveals that the assumption of equalizing the solid conveying angle with the groove angle is appropriate in this case. To obtain proper results, it is also necessary to take into account the bulk density as a function of dumping height.

### 3.3. Results of the Solid Conveying Behavior of PP Granules

**Figure 8:**
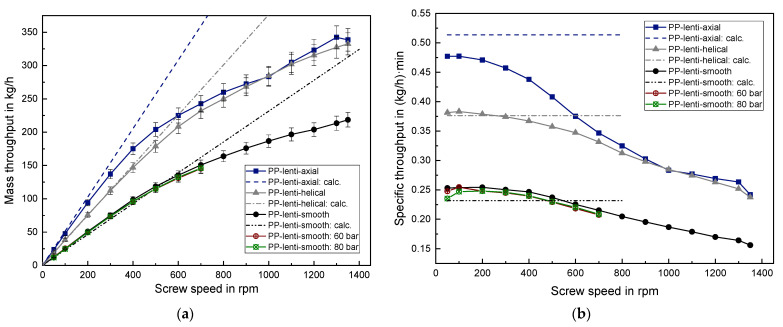
(**a**) Absolute mass throughput and (**b**) specific mass throughput for lenticular PP.

**Figure 9:**
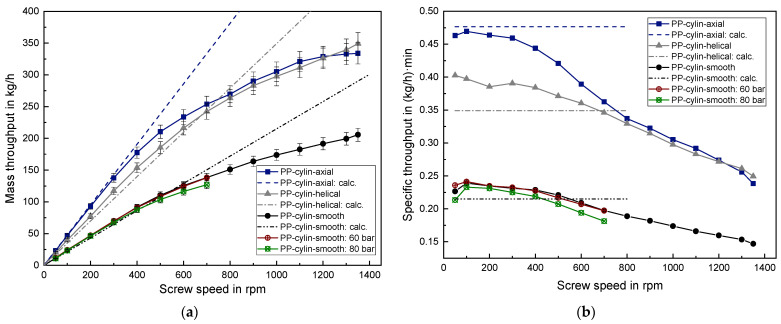
(**a**) Absolute mass throughput and (**b**) specific mass throughput for cylindrical PP.

The results of processing lenticular PP and cylindrical PP can be found in Figure 8 and Figure 9, respectively. The qualitative behavior is analogous to the previous findings of PE-HD, meaning that the axially grooved barrel results in the highest mass throughput at low screw speed, but approaches the values of the helically grooved barrel at higher screw speeds. The linear behavior converts into a degressive behavior for both PP granule geometries if a threshold screw speed of roughly 300 rpm is exceeded. A significant influence of the barrel type as well as of the granule geometry on the threshold screw speed cannot be observed. The average grain mass of cylindrical PP is 27.8 mg which is nearly identical to the average grain mass of lenticular PP with 29.2 mg.

Applying a back pressure onto the solid conveying zone led only to a vanishingly small mass throughput reduction for both PP granules with a slightly higher reduction for 80 bar compared to 60 bar, in contrast to the observations of PE-HD. This reveals that not only the granule geometry but also the type of plastic strongly influences the back pressure dependency in the solid conveying zone. In order to properly account for this, the compressibility of the different granules should be considered in future research as a further variable. A description of an experimental and a simulative approach to determine the compressibility can be found in [17].

In the case of lenticular PP, the analytical predictions result in a mean deviation of around 8% for the smooth barrel, around 9% for the axially grooved barrel and only 1% for the helically grooved barrel, considering the low screw speed range from 50–300 rpm. Again, using the helically grooved barrel leads to an extremely well mass throughput prediction, whereas the calculations for the axially grooved barrel overpredict the mass throughput.

The cylindrical PP is an exception in the speed range from 50–300 rpm since the measured mass throughput coincides well with the predicted throughput for the axially grooved barrel with a relative deviation of only 3%. Contrary to this, in the helically grooved case the calculation underestimates the measured throughput by around 11%. The exact reason for this quite high deviation in the helically grooved case cannot be given. However, it can be assumed that the measurement of the bulk density for the cylindrical PP is error-prone to a certain extent. Thus, the real bulk density is expected to be higher than the measured bulk density. The granule size distribution of the virgin lenticular PP is notably smaller compared to the broad size distribution of the regranulated cylindrical PP. Hence, it can be assumed that measuring the bulk density for regranulates is more susceptible to error compared to uniform virgin granules. This observation emphasizes the importance of a careful determination of the bulk density as a prerequisite for enabling an accurate mass throughput prediction, especially for granules which possess a broad size distribution.

### 3.4. Results of the Solid Conveying Behavior of PA Granules

Processing the spherical PA and the cylindrical PA with the helically grooved barrel led to a very high extruder torque which exceeded the permissible motor power. Hence, both series of experiments could not be performed even at low screw speeds. 

**Figure 10:**
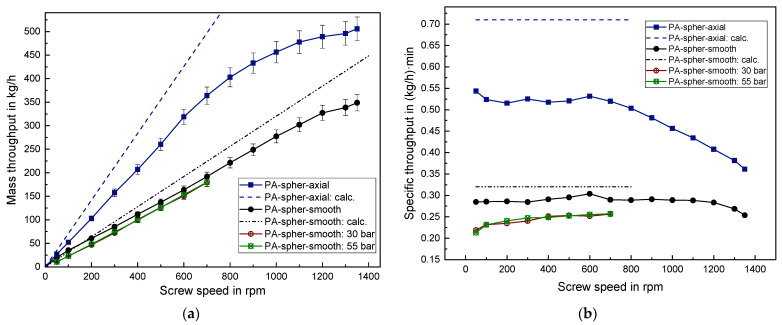
(**a**) Absolute mass throughput and (**b**) specific mass throughput for spherical PA.

Moreover, a high mechanical wear occurred at the cone of the back pressure element when processing the spherical PA because PA exhibits a significantly higher hardness compared to the previously examined polyolefins. Hence, applying a back pressure was omitted in the case of cylindrical PA to avoid further damage. Due to the previous explanation the maximum back pressure was set to 55 bar for the spherical PA. This resulted in a mean decrease in mass throughput of around 14–18% for both 30 bar and 55 bar compared to experiments without back pressure (see Figure 10).

**Figure 11:**
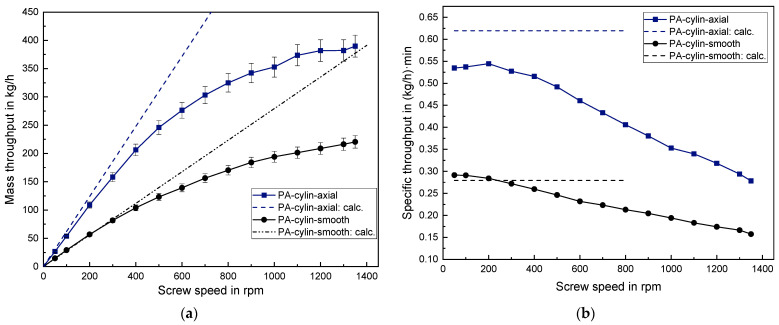
(**a**) Absolute mass throughput and (**b**) specific mass throughput for cylindrical PA.

Spherical PA results in a very large mass throughput with around 505 kg/h at 1350 rpm. In the case of an axially grooved barrel a linear behavior is observed until 600 rpm with the specific throughput remaining constant at around 0.52 (kg/h)·min. Using the smooth barrel even results in a linear behavior until 1200 rpm with a specific throughput of around 0.29 (kg/h)·min. The cylindrical PA exhibits an early onset of degressive behavior at 300 rpm for both the smooth and the axially grooved barrel as can be seen Figure 11. This is contrary to the late onset of non-linearity when processing the spherical PA. Thus, the small spherical PA particles with an average grain mass of 12.4 mg possess a considerably advantageous trickling behavior into the screw channels below the hopper, compared to the larger cylindrical PA particles with an average grain mass of 26.7 mg.

When processing the spherical PA in the smooth barrel, the prediction quality is very good with only 3% relative deviation between the calculated and the measured throughput at screw speeds between 50–300 rpm. With regard to the axially grooved barrel, however, the prediction quality is very poor with a relative deviation of about 35%. Nevertheless, even though the calculations for the axially grooved barrel usually exceeded the measured data in the previous cases, this deviation is the highest deviation observed in this work. This can be explained by the fact that applying Equations (5) and (7) assumes a nut-screw conveying, which resembles the solid conveying case 1.(a). However, this assumption is not applicable to the spherical PA since the ESD of 2.68 mm is smaller than the groove depth of 2.8 mm. Hence, to improve the prediction quality the solid conveying case 2.(a) should be used (see Chapter 1.2). This would exclude the additional mass flow in the grooves but still contain a friction increase to the barrel due to the grooves filled with plastic granule and thus a variation of the solid conveying angle.

### 3.5. Comparing the Results of the Mere Solid Conveying Zone to an Entire Extruder Set-Up including Melting

**Figure 12:**
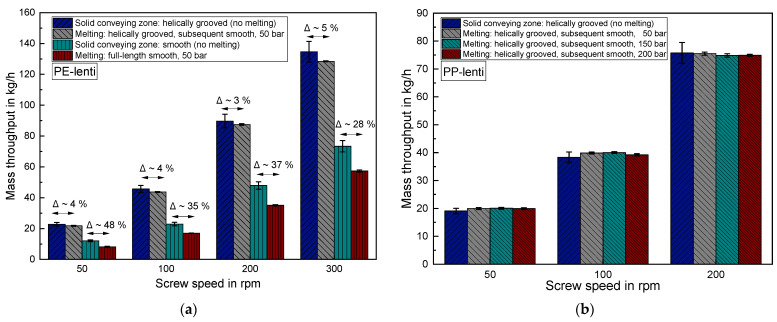
(**a**) Comparing the lenticular PE throughput in the solid conveying zone to the throughput in a whole extrusion set-up with helically grooved solid conveying zone and smooth barrel in the melting and metering zone for 50 bar throttle die back pressure. (**b**) Comparing the lenticular PP throughput in the helically grooved solid conveying zone to the throughput in a whole extrusion set-up with helically grooved solid conveying zone and smooth barrel in the melting and metering zone for 50 bar, 100 bar and 200 bar throttle die back pressure.

Figure 12a shows the mass throughput results when processing the lenticular PE with an entire extruder set-up, which also includes melting of the granules. The throttle die was set to a die back pressure of 50 bar. Comparing the results of the helically grooved solid conveying zone to the entire extruder set-up reveals only a minor deviation of around 3–5% for all screw speeds at 50 bar die back pressure. Hence, the previously shown results of the mere grooved solid conveying zones are well transferable to real extrusion processes which also include melting.

As opposed to this, the results of the smooth solid conveying zone and the full-length smooth extruder differ significantly between 28–48%. The results of the mere solid conveying zone are thus not transferable to entire extrusion processes when a smooth barrel is utilized. In this case, the measured values are always substantially lower for the entire extrusion set-up because of a pronounced counter pressure dependent conveying behavior of smooth barrel systems [1].

The results of melt processing the lenticular PP with the helically grooved entire extruder set-up are depicted in Figure 12b for an increasing throttle die back pressure from 50 bar to 200 bar. Again, the results of the mere grooved solid conveying zone perfectly match with the results of mass throughput determination of the entire extruder set-up. Moreover, increasing the throttle die back pressure has no effect on the mass throughput. This finding confirms the counter pressure independent behavior of the helically grooved systems. It should be mentioned that this finding only applies up to a certain threshold counter pressure which varies depending on the respective extruder design and processed material. If this threshold is exceeded, it is also possible for observing a decreasing throughput in grooved systems with increasing counter pressure [1]. 

## 4. Conclusions and Outlook

### 4.1. Conclusions

In this study, the solid conveying behavior of different plastic granule geometries was investigated experimentally for the first time using a smooth as well as two grooved barrels up to screw speeds of 1350 rpm. It was found that the shape and size of the plastic granule play a decisive role in determining the start of a degressive mass throughput behavior. A small and spherical granule exhibited an advantageous behavior compared to larger lenticular and cylindrical shaped granules. In the case of the spherical granule, the threshold screw speed is 1200 rpm for a smooth barrel and 600 rpm for an axially grooved barrel. In the case of the lenticular and the cylindrical granules the threshold screw speed is only around 300 rpm for both smooth and grooved barrels.

Moreover, it was revealed for the first time that the mass throughput equalizes for an axially and a helically grooved solid conveying zone at screw speeds higher than approximately 800 rpm. This is contrary to the low screw speed range where the axially grooved barrel results in a significantly higher throughput than the helically grooved barrel. Thus, the maximum throughput at high screw speeds is limited by the granule stream provided by the hopper opening and is no longer governed by the groove angle respectively the associated solid conveying angle.

Furthermore, it was shown that using a simple linear calculation approach and assuming a nut-screw conveying results in a proper prediction for the helically grooved solid conveying zone at low screw speeds. However, this applies only if the granule possesses a diameter that is larger than the groove depth and if the screw channel depth is smaller than two times the granule diameter (see solid conveying case 1.(a)). Contrary to the accurate prediction in the helically grooved case, using the linear approach always led to an overestimation for the axially grooved solid conveying zone since the actual solid conveying angle in the screw channels is somewhat lower than 90°.

A full-length smooth extruder notably differed in the mass throughput compared to the mere smooth solid conveying zone. As opposed to this, the mass throughput for an entire extruder with a grooved feed zone and a subsequently smooth melting and metering zone was in very good accordance with the results of the mere grooved solid conveying zone. This confirms the counter pressure independent behavior of these grooved set-ups. 

### 4.2. Outlook

As a next step, a regrind material will be investigated in future work to work out whether using the linear calculation approach, which assumes spherical granules, still holds for plastics with a plate-shaped geometry. Moreover, plastic powder will be examined to investigate whether the late start of degressive throughput behavior is also found when processing spherical materials with a very small particle size. Furthermore, to appropriately incorporate the non-linear behavior at high screw speeds, the obtained experimental results will be used in following work to establish a new analytical calculation model. This new model shall be based solely on physically meaningful parameters such as the average granule mass, the aspect ratio or the pourability of the granule. One possible way could be a coupling of the linear approach with a proper function of limited growth, to account for effects such as a vortex formation in the hopper.

Besides this proceeding the solid conveying problem in a grooved barrel can also be addressed via numerical simulation using the discrete element method (DEM) which appears to be another promising way. This DEM approach was previously examined in [17,38] for low screw speeds up to 100 rpm and recently thoroughly studied in [39] for screw speeds up to 500 rpm.

## Figures and Tables

**Table 1:** Geometrical plastic granule dimensions.

Granule	Height *h* in mm	Diameter *d*_1_ in mm	Diameter *d*_2_ in mm	Equivalent Spherical Diameter (ESD) in mm	Average Grain Mass in mg
PE-HD					
Cylindrical	2.81 ± 0.10	3.22 ± 0.10	3.91 ± 0.10	3.76	28.6
Lenticular	1.96 ± 0.05	4.40 ± 0.16	4.69 ± 0.09	3.93	35.2
PP					
Cylindrical	2.88 ± 0.40	2.86 ± 0.21	4.43 ± 0.19	3.79	27.8
Lenticular	2.28 ± 0.01	3.70 ± 0.07	4.35 ± 0.04	3.8	29.2
PA					
Cylindrical	2.85 ± 0.11	3.14 ± 0.17	3.25 ± 0.20	3.52	26.7
Spherical	2.34 ± 0.06	2.67 ± 0.11	3.08 ± 0.31	2.68	12.4

**Table 2:** Geometrical dimensions of the screw and of the helically and axially grooved barrel.

Geometry Parameters	Dimension
Outer screw diameter $D_{s}$	34.85 mm
Core diameter of the screw $D_{c}$	23.85 mm
Helix angle of the screw φ	17.73°
Screw channel depth $h_{s}$	5.5 mm
Width of the screw flight $w_{f}$	3.5 mm
Number of screw flights $i_{s}$	1
Width of a groove $w_{g}$	5.5 mm
Groove angle ω	41.19° (helical) 90.00° (axial)
Groove depth $h_{g}$	2.8 mm
Number of grooves $i_{g}$	6 (helical) 10 (axial)

**Table 3:** Bulk density fitting parameters for all used plastic granules based on Equation (4).

Granule	Bulk Density Fit Parameter				Bulk Density at 5.5 mm Dumping Height in g/cm^3^
	$\rho_{0}\;\mathrm{in}\;g/{\mathrm{cm}}^{3}$	$h_{0}\;\mathrm{in}\;\mathrm{mm}$	*A* (Dimensionless)	*B* (Dimensionless)	
PE-HD					
Cylindrical	0.529	1.995	1.505	0.529	0.459
Lenticular	0.582	2.022	1.596	0.283	0.492
PP					
Cylindrical	0.491	1.787	1.122	0.463	0.389
Lenticular	0.531	1.923	1.203	0.414	0.419
PA					
Cylindrical	0.62	1.986	1.366	0.372	0.506
Spherical	0.69	0.208	0.381	0.485	0.579

## Data Availability

Not applicable.
